# Two Cellular Protein Kinases, DNA-PK and PKA, Phosphorylate the Adenoviral L4-33K Protein and Have Opposite Effects on L1 Alternative RNA Splicing

**DOI:** 10.1371/journal.pone.0031871

**Published:** 2012-02-21

**Authors:** Heidi Törmänen Persson, Anne Kristin Aksaas, Anne Katrine Kvissel, Tanel Punga, Åke Engström, Bjørn Steen Skålhegg, Göran Akusjärvi

**Affiliations:** 1 Department of Medical Biochemistry and Microbiology, Uppsala University, Uppsala, Sweden; 2 Department of Nutrition, University of Oslo, Oslo, Norway; Tel Aviv University, Israel

## Abstract

Accumulation of the complex set of alternatively processed mRNA from the adenovirus major late transcription unit (MLTU) is subjected to a temporal regulation involving both changes in poly (A) site choice and alternative 3′ splice site usage. We have previously shown that the adenovirus L4-33K protein functions as an alternative splicing factor involved in activating the shift from L1-52,55K to L1-IIIa mRNA. Here we show that L4-33K specifically associates with the catalytic subunit of the DNA-dependent protein kinase (DNA-PK) in uninfected and adenovirus-infected nuclear extracts. Further, we show that L4-33K is highly phosphorylated by DNA-PK *in vitro* in a double stranded DNA-independent manner. Importantly, DNA-PK deficient cells show an enhanced production of the L1-IIIa mRNA suggesting an inhibitory role of DNA-PK on the temporal switch in L1 alternative RNA splicing. Moreover, we show that L4-33K also is phosphorylated by protein kinase A (PKA), and that PKA has an enhancer effect on L4-33K-stimulated L1-IIIa splicing. Hence, we demonstrate that these kinases have opposite effects on L4-33K function; DNA-PK as an inhibitor and PKA as an activator of L1-IIIa mRNA splicing. Taken together, this is the first report identifying protein kinases that phosphorylate L4-33K and to suggest novel regulatory roles for DNA-PK and PKA in adenovirus alternative RNA splicing.

## Introduction

**Figure 1:**
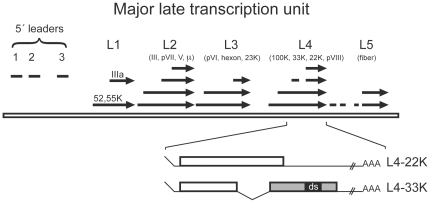
Schematic drawing showing the spliced structure of the major late transcription unit. The tri-partite leader is labelled 5′ leaders (to the left), which is included in all mRNAs expressed from the MLTU. The arrows show the different mRNAs from each family unit (L1–L5) produced during infection. The 52,55K mRNA from the L1 unit is the only mRNA produced early during infection. Below, the organisation of the L4-22K and L4-33K open reading frames including the functional important ds region.

Gene expression is a highly sophisticated molecular machinery involving a coordinated action of multiple proteins. Human adenovirus has been a popular model system to elucidate the interplay between different proteins and gene expression mechanisms. Indeed, adenovirus-based studies have contributed to the general understanding of the basic gene expression mechanisms such as gene transcription, pre-mRNA splicing and mRNA export. Adenovirus gene expression is regulated in a highly coordinated manner during the infectious cycle. Hence, the adenovirus genes are sequentially expressed during the infection, producing regulatory proteins directly following the infection (early proteins) and structural proteins after the onset of viral genome replication (late proteins). The collection of late proteins (at least 15 different proteins) is encoded from a single precursor RNA (pre-mRNA) originating from the, so-called, major late transcription unit (MLTU). The MLTU produces five families of mRNAs with co-terminal poly (A) sites (L1–L5, Fig. 1). Following the selection of poly (A) site the pre-mRNA is spliced to generate a minimum of 20 alternatively spliced mRNAs, which all have a common 201 nucleotide long tripartite leader sequence at their 5′ end and diverse 3′ end sequences. L1 is the only unit in MLTU producing mRNAs both early and late after infection. The last intron in L1 is spliced using a common 5′ splice site and two alternative 3′ splice sites to produce two mRNAs, the 52,55K mRNA (proximal 3′ splice site), and the IIIa mRNA (distal 3′ splice site). L1 mRNA expression is subjected to a temporal regulation. Thus, the 52,55K mRNA is produced both early and late after infection while IIIa mRNA is only produced late. The amazing encoding variability of adenovirus gene expression is the result of coordinated action of viral and host cell proteins on regulatory mechanisms occurring at the transcriptional and post-transcriptional level [1].

The L4 unit encodes for a minimum of four mRNAs (Fig. 1), of which two are of interest for this study. They encode for two related proteins; L4-22K and L4-33K (Fig. 1). Work from our group and others have shown that both proteins are key regulators of adenovirus late gene expression by targeting pre-mRNA splicing and MLTU transcription [2]-[5]. The L4-33K and L4-22K proteins share a common N-terminus, but have unique carboxyl-terminal domains. Both proteins have also been suggested to perform similar functions, including packaging of the viral genome and binding to major late promoter sequences [6]–[8]. We have previously characterized the adenovirus L4-33K protein as a novel factor regulating pre-mRNA splicing in human cells. It functions as a key activator of the L1 alternative splicing inducing the production of L1-IIIa mRNA in uninfected cells [2]. The splicing activation domain was mapped to the highly conserved carboxyl-terminal ds region (Fig. 1) containing a tiny RS-repeat.

DNA-dependent protein kinase (DNA-PK) is a nuclear serine/threonine protein kinase that belongs to the family of phosphatidylinositol 3-kinase-like kinases (PIKKs) [9]. Phosphorylation of most DNA-PK substrates, like p53, is activated by linear double stranded DNA (dsDNA) [10]–[12]. Biochemical studies have shown that DNA-PK is a heterotrimeric enzyme composed of a catalytic subunit (DNA-PKcs) and two regulatory subunits Ku86 and Ku70 [13], [14]. DNA-PK is an essential protein directly involved in the double strand break repair system (DSBR) pathway [15]. To repair DNA double strand breaks (DSB), the Ku heterodimer recognizes the DSB and facilitates the recruitment of DNA-PKcs and the rest of DSBR pathway components to the injured DNA [10]–[12].

Beside the involvement in DNA repair, DNA-PK has also been suggested to have a direct role in transcription. DNA-PK interacts with RNA polymerase II (RNAPII) and phosphorylates the C-terminal domain (CTD) of RNAPII, thereby regulating the transcription initiation step [16]–[18]. Also, DNA-PK has a regulatory role in transcription by phosphorylating transcription factors like Sp1, Oct-1, c-Myc, c-Jun, p53 and thereby regulating their functions [19].

It is well established that several protein kinases are involved in the regulation of gene transcription. This includes cAMP dependent protein kinase (PKA), which regulates gene expression by phosphorylating several transcription factors, including the cAMP- responsive element (CRE) binding protein (CREB) [20]. In the absence of cAMP, PKA is an inactive tetramer composed of a regulatory (R) subunit dimer and two catalytic (C) subunits. PKA subunits are encoded by four R and C subunit genes, respectively (RIα, RIIα, RIβ, RIIβ, Cα, Cβ, Cγ and PRKX) [21]. Specificity in the cAMP/PKA signal transduction pathway is obtained by tissue-specific expression and targeting the R subunits to A-kinase anchoring proteins (AKAPs) in the cytosol and targeting of the C subunit to C subunit-binding proteins both in the cytosol and nucleus [22]–[28]. PKA is activated upon binding of four molecules of cAMP to the R dimer, releasing the C subunits to phosphorylate relevant substrates on serine and threonine residues in its vicinity [29]. A proportion of the C subunits translocates to the nucleus after activation [30], [31]. In addition to gene transcription, others and we have demonstrated that nuclear PKA regulates pre-mRNA splicing through phosphorylation of splicing factors, such as Serine/arginine-rich splicing factor 1 (SRSF1) and interaction with Splicing factor arginine/serine-rich 17A (SFRS17A) [32]–[34]. Nuclear C subunits are inhibited and transported out of the nucleus by the heat-stable protein kinase inhibitor (PKI) [35].

Here we show that the adenovirus L4-33K protein specifically associates with the DNA-PKcs in uninfected and adenovirus-infected nuclear extracts. Interestingly, the L4-33K protein is highly phosphorylated by DNA-PK *in vitro* in a double stranded DNA-independent manner. Importantly, DNA-PK deficient cells show enhanced production of the L1-IIIa mRNA suggesting an inhibitory role of DNA-PK on the temporal switch in L1 alternative RNA splicing. Furthermore, we show that L4-33K is phosphorylated by PKA, and that PKA stimulates L4-33K-induced L1-IIIa splicing. Taken together, our data suggests that both DNA-PK and PKA phosphorylate L4-33K, regulate RNA splicing and have opposite effects on adenovirus alternative RNA splicing.

## Results

### Proteomic analysis of L4-33K interacting proteins

**Figure 2:**
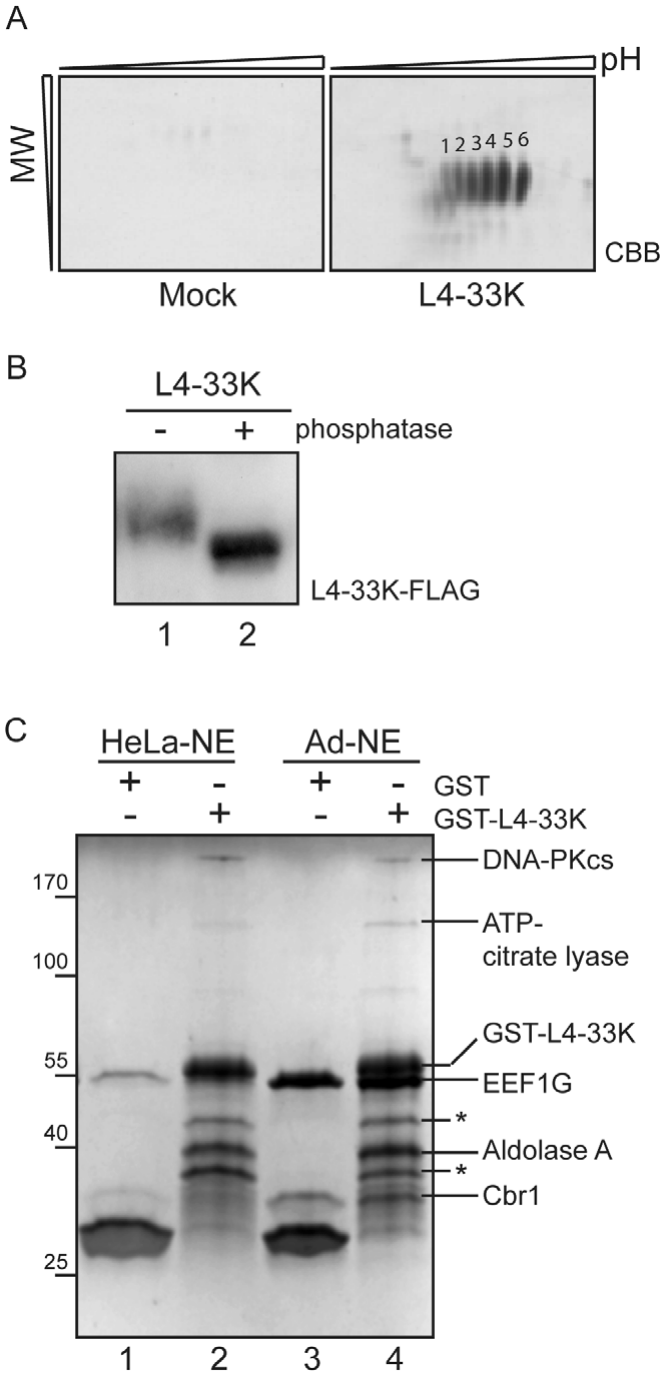
Proteomic analysis of L4-33K and interacting proteins. A) Immuno-purified complexes from HEK293 cells transfected with either empty control plasmid (Mock) or L4-33K-Flag expressing plasmid (L4-33K) resolved on 2D-gel electrophoresis (first dimension pH 3–11 and second dimension 12.5% SDS-PAGE) and stained with colloidal CBB. Numbers 1–6 point out the six spots identified by mass spectrometry analysis as different isoelectric forms of L4-33K. B) HEK293T cells were transfected with L4-33K-FLAG. Cell lysates were adjusted to equal protein concentration, precleared with magnetic beads before immunoprecipitation using anti-FLAG rabbit and magnetic beads. Lysates were left untreated or treated with alkaline phosphatase (−/+ phosphatase) for 30 minutes before analysis by immunoblotting using anti-FLAG M2. C) Pull-down with Glutathione coated beads was performed on HeLa-nuclear extracts (HeLa-NE) or adenovirus infected NE (Ad-NE) incubated with bacterially expressed and purified GST-L4-33K or GST proteins (see Material and Methods). Pulled-down proteins from HeLa NE (lanes 1 and 2) and Ad-NE (lanes 3 and 4) were separated on SDS-PAGE (Biorad, Any kD™) followed by staining with colloidal CBB. All visible bands were cut out and identified by mass spectrometry analysis to be DNA-dependent protein kinase catalytic subunit (DNA-PKcs, 470 kDa), ATP-citrate lyase (121 kDa), eukaryotic translation elongation factor 1 gamma (EEF1G, 50 kDa), Aldolase A (40 kDa) and Carbonyl reductase (Cbr1, 30 kDa). The asterix (*) indicates fusion protein degradation.

Adenovirus L4-33K is a multifunctional phosphoprotein suggested to be involved in several aspects of virus gene expression [2], [4], [5], [36], [37]. Despite the characterized functions, little is known about the extent of the post-translational modifications of L4-33K. To study the complexity of post-translational modifications we transfected HEK293 cells with an L4-33K-FLAG expressing plasmid or an empty control plasmid (Mock) and studied the protein profile by 2D gel electrophoresis of immuno-purified complexes. As shown in Fig. 2A, L4-33K resolved into six major spots with approximately the same molecular weight but different pI values. The identity of the individual spots as L4-33K was confirmed by mass spectrometry analysis. In conclusion, this experiment suggests that L4-33K exist in several different isoelectric forms in cells, modifications that most likely, at least to some extent, are due to varying levels of phosphorylation of the protein. To validate that L4-33K is a phosphoprotein *in vivo* HEK293T cells were transfected with L4-33K-FLAG and subjected to a dephosphorylation assay. Immunoprecipitated L4-33K-FLAG was left untreated or treated with alkaline phosphatase (Fig. 2B). This resulted in a size reduction corresponding to dephosphorylation of L4-33K, confirming that L4-33K is a phosphoprotein *in vivo*.

To obtain a better mechanistic understanding of L4-33K activities, we were interested in identifying proteins potentially interacting with the L4-33K protein. For this purpose we used a Glutathione-S-Transferase (GST) pull-down assay system. Purified GST-L4-33K fusion protein was incubated with uninfected or adenovirus-infected HeLa nuclear extracts. The nuclear proteins, interacting with the fusion protein, were visualized by CBB staining after SDS-PAGE (Fig. 2C) and identified by mass spectrometry sequencing. In total we identified five proteins specifically interacting with L4-33K under our experimental conditions (Fig. 2C). Two of the proteins, eukaryotic translation elongation factor 1 gamma (EEF1G) and Carbonyl reductase 1 (Cbr1), contain GST domains in their protein sequence and were therefore regarded as potential false positive interactors [38], [39]. We also identified two proteins with assigned functions in host cell metabolism: ATP-citrate lyase and Aldolase A. Interestingly, the L4-33K protein specifically interacted with the catalytic subunit of the double stranded DNA-dependent protein kinase (DNA-PKcs), both in uninfected and adenovirus infected HeLa cell nuclear fractions (Fig. 2C). Since L4-33K is a phosphoprotein with a suggested function as an alternative splicing factor and DNA-PK has been connected to transcriptional regulation, which in turn is known to regulate alternative splicing we decided to focus our initial efforts and further explore the potential interplay between L4-33K and DNA-PK.

### L4-33K interacts with DNA-PKcs during a lytic infection

**Figure 3:**
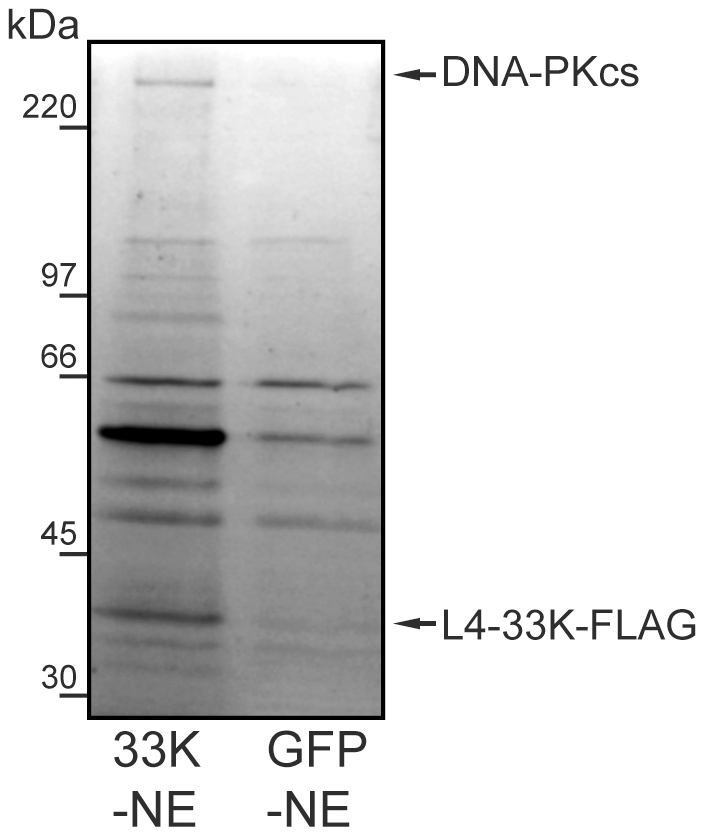
DNA-PK co-immunoprecipitates with L4-33K. Nuclear extracts (NE) derived from AdEasy-L4-33K (33K-NE, lane 1) and AdEasy-GFP (GFP-NE), infected HEK293 cells were Anti-FLAG M2-agarose affinity gel purified using 3×FLAG peptide-eluted preparation and analysed by SDS-PAGE in 12% gels followed by staining with colloidal CBB. All stained bands were cut out and analysed by mass spectrometry.

The initial identification of DNA-PKcs as a L4-33K associating protein urged us to further validate this interaction under more relevant *in vivo* conditions. For this purpose we constructed a recombinant AdEasy virus expressing a FLAG-epitope tagged L4-33K protein under the control of a tetracycline inducible promoter (AdEasy-L4-33K). HEK293 cells were infected with the AdEasy-L4-33K virus or an AdEasy-GFP control virus and nuclear extracts prepared at 20 hpi. In this experiment we did not induce L4-33K-FLAG expression with doxycycline, since our preliminary experiments had shown that high level of L4-33K protein expression was toxic to cells and resulted in an aberrant subcellular localization of the L4-33K protein (data not shown). It should be noted that there is a detectable level of background L4-33K expression at 20 hpi, in the absence of inducer. This leakage most likely results from the increase in available DNA templates for transcription resulting from viral DNA replication. As shown in Fig. 3, immunoprecipitation of the L4-33K-FLAG protein revealed a specific co-purification of a high molecular weight protein on SDS-PAGE that by mass spectrometry sequencing was shown to be DNA-PKcs. Based on these results we conclude that L4-33K interacts with DNA-PKcs both *in vitro* (Fig. 2C) and *in vivo* during a lytic virus infection (Fig. 3).

### L4-33K is phosphorylated by DNA-PK in a dsDNA-independent manner

**Figure 4:**
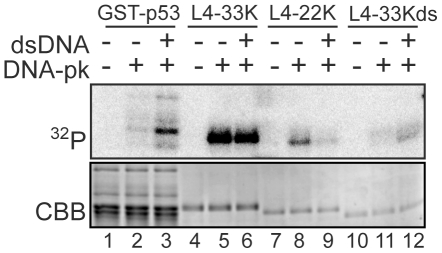
L4-33K is phosphorylated by DNA-PK in a DNA-independent manner. GST-p53 (lanes 1–3) and His-tagged L4-33K (lanes 4–6), L4-22K (lanes 7–9) and L4-33Kds were incubated in the presence (+) and absence (−) of DNA-PK and γ^32^P ATP in the presence (+) and absence (−) of the DNA-PK activator, linear double stranded DNA (dsDNA). Phosphorylated proteins, detected as ^32^P-labelled, were resolved by SDS-PAGE in 12% gels and incorporation of radiolabelled phosphate visualised by autoradiography (upper panel). Equal loading of each lane was verified by CBB staining (lower panel).

Since DNA-PK is a protein kinase we investigated whether the L4-33K protein was a substrate for DNA-PK phosphorylation. For this experiment we performed *in vitro* kinase assays with purified L4-33K and DNA-PK. As shown in Fig. 4, L4-33K was efficiently phosphorylated by DNA-PK (lane 5). Interestingly, DNA-PK phosphorylation of L4-33K was not enhanced by addition of dsDNA to the reaction mixture (lanes 5 and 6). Phosphorylation of most DNA-PK substrates, like p53 (lanes 2 and 3), is activated by dsDNA (see Discussion). This finding suggests that L4-33K is phosphorylated by DNA-PK in an unusual dsDNA-independent manner. Since the L4-33K and its relative L4-22K proteins share a common N-terminus but have unique C-terminal ends (Fig. 1) we decided to test if the L4-22K protein also was a substrate for DNA-PK. As shown in Fig. 4, L4-22K was a poor substrate, compared to L4-33K, for DNA-PK phosphorylation (lanes 5–9). However, of some interest, the low level of L4-22K phosphorylation was not activated, but inhibited by addition of dsDNA (lanes 8 and 9).

The splicing enhancer function of L4-33K was previously mapped to the ds region of L4-33K (Fig. 1), which contains a tiny RS repeat that we previously showed is important for function [2]. Since splicing factors often are regulated by phosphorylation we tested whether an L4-33K protein lacking the ds region, L4-33Kds, was a substrate for DNA-PK phosphorylation. As shown in Fig. 4 (lanes 11 and 12) L4-33Kds was a poor substrate compared to both the wild type L4-33K (lane 5) and L4-22K (lane 8) proteins. Surprisingly, this low level of phosphorylation was enhanced by dsDNA (lanes 11 and 12).

### DNA-PK inhibits the shift in L1 alternative RNA splicing

**Figure 5:**
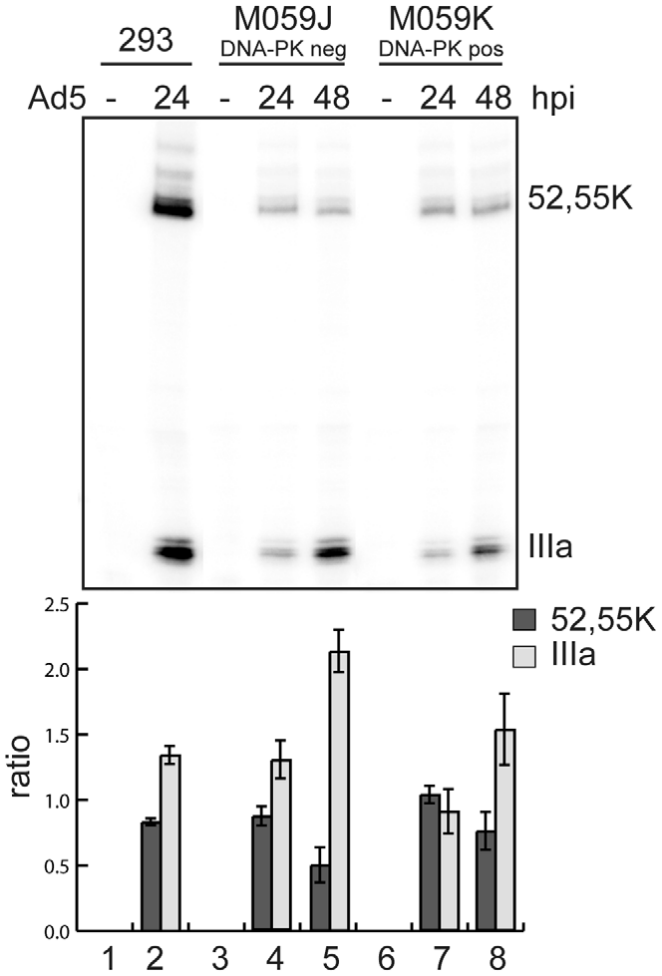
DNA-PK reduces the accumulation of L1-IIIa mRNA during lytic infection. HEK293 cells (293, lanes 1 and 2), M059J cells (DNA-PKcs negative, M059J, lanes 3–5)) and M059K cells (DNA-PKcs positive, M059K, lanes 6–8) were infected with wild type Ad5 at 10 FFU/cell and cytoplasmic RNA was harvested from uninfected (−) or infected cells at 24- and 48 h pi. L1 mRNA expression was measured by S1 protection assay as described in Material and Methods. Detection of L1-52,55K and L1-IIIa mRNAs are marked. The relative levels of L1-52,55K and L1-IIIa mRNAs were quantified and plotted as the ratio of L1-52,55K or L1-IIIa mRNA over total L1 mRNAs and normalized against the 24 h infection of MO59K cells producing the ratio (lower panel) (five experiments, p<0.05, t-test).

Since DNA-PK phosphorylated L4-33K we considered the possibility that DNA-PK may function as a regulator of adenovirus alternative splicing. To test this hypothesis we analyzed L1 mRNA expression in the DNA-PKcs deficient cell line, MO59J, and its wild type counterpart, MO59K cells. The MO59J and MO59K cells were infected with wild-type Ad5, cytoplasmic RNA isolated at 24 and 48 hpi and the production of the L1-52,55K (early pattern) and L1-IIIa (late pattern) of mRNAs monitored by the S1 nuclease protection assay (Fig. 5). Interestingly, we detected a significant increase (p<0.05, t-test) in the accumulation of the L1-IIIa mRNA in the DNA-PK deficient cell line, MO59J (Fig. 5), suggesting that DNA-PK has a negative effect on the switch from the early to the late pattern of L1 alternative splicing.

### L4-33K is phosphorylated by PKA

**Figure 6:**
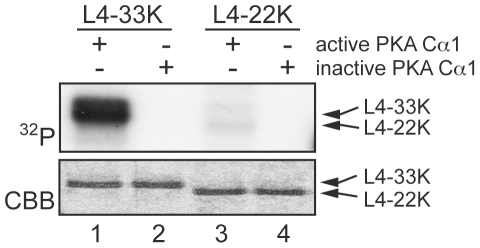
Phosphorylation of L4-33K by PKA. Purified L4-33K (lanes 1–2) and L4-22K (lanes 3–4) were incubated with active or heat-inactivated PKA Cα1 and γ-^32^P-ATP in the reaction buffer. Protein samples were analyzed by SDS-PAGE followed by CBB staining (lower panel) and autoradiography (upper panel).

Based on previous reports that PKA phosphorylates certain splicing factors we wanted to test if L4-33K is a PKA substrate. For this experiment we incubated active or heat inactivated PKA Cα1 with either purified L4-33K or L4-22K. As shown in Fig. 6, L4-33K but not L4-22K was efficiently phosphorylated by active PKA Cα1 *in vitro* (lane 1 and 3).

### PKA enhances L4-33K activated L1 alternative splicing

**Figure 7:**
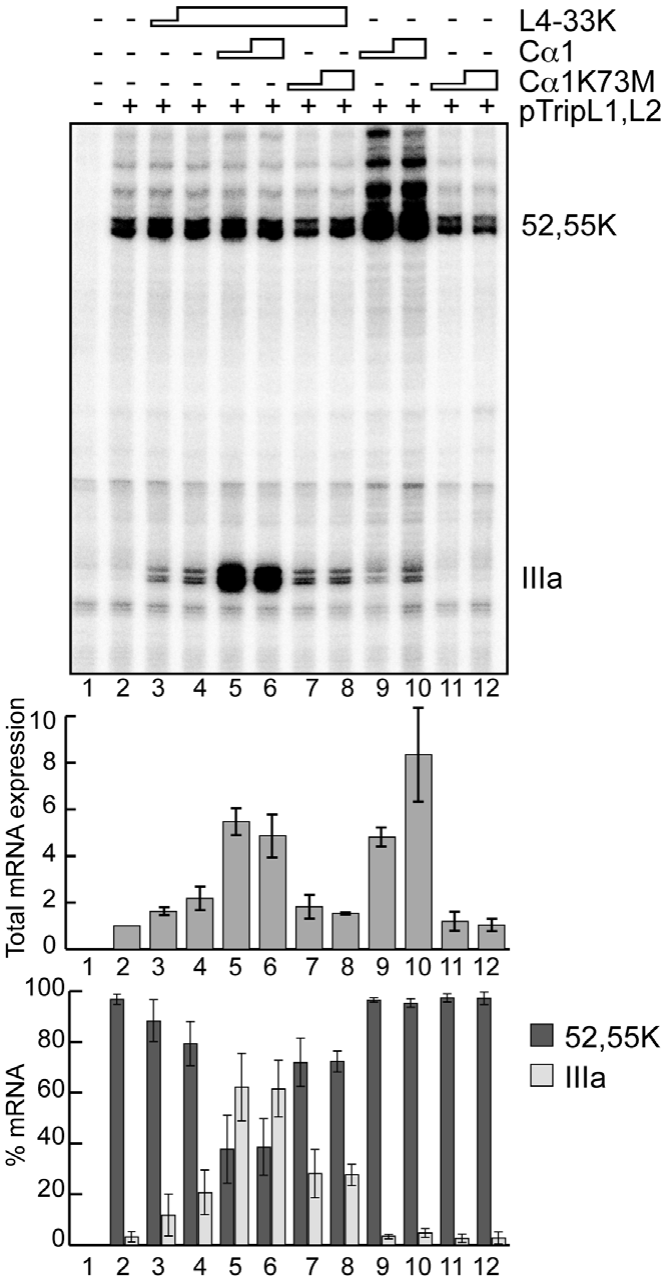
Additive effect of PKA on L4-33K stimulated IIIa splicing. HEK293T cells were left untreated (lane 1) or transfected with reporter plasmid pTripL1,L2 (lanes 2–12) and co-transfected with L4-33K alone (lanes 3–4) or L4-33K in conjunction with either PKA Cα1 (lanes 5–6) or Cα1K73M (lanes 7–8). As controls HEK293T cells were transfected with either Cα1 (lanes 9–10) or Cα1K73M (lanes 7–8) without L4-33K (lanes 9–12). Total RNA was isolated and L1 mRNA expression measured by S1 protection assay as described in Material and Methods. Detection of L1-52,55K and L1-IIIa mRNAs are marked. Total L1 mRNA was quantified and plotted as the ratio of total L1 mRNA over the total mRNA in lane 2 (only transfected with pTripL1,L2 plasmid). The levels of L1-52,55K and L1-IIIa mRNAs were quantified and plotted as the ratio of L1-52,55K or L1-IIIa mRNA over total L1 mRNAs (% mRNA). Average transcription and splicing efficiencies with standard deviations are shown (three experiments).

Based on these observations and the fact that the PKA catalytic subunit is involved in pre-mRNA splicing [32]–[34] we investigated whether PKA regulates L4-33K-dependent L1 alternative splicing in an *in vivo* transient co-transfection assay. For this experiment HEK293T cells were co-transfected with a reporter plasmid expressing the L1 gene and plasmids expressing PKA Cα1 or a kinase inactive mutant, Cα1K73M, in the absence or presence of an L4-33K expressing plasmid. Protein expression was confirmed by immunoblotting using antisera detecting L4-33K or the PKA C subunit (data not shown). The accumulation of L1 mRNAs was analyzed by the S1 protection assay. As expected from our previous results [2], L4-33K activated L1-IIIa mRNA accumulation (Fig. 7, lanes 2–4). Transfection of Cα1 alone resulted in a marked increase in total L1 mRNA accumulation (Fig. 7 middle panel, compare lanes 2, 9 and 10), suggesting that PKA activates transcription from the adenovirus MLP. However, it is noteworthy that Cα1 transfection did not change the ratio of L1-52,55K/IIIa mRNA accumulation (Fig. 7, lower panel, lanes 9 and 10). In contrast, co-transfection of Cα1 together with L4-33K resulted in both the increase in total L1 mRNA accumulation (Fig. 7, middle panel), seen with Cα1 alone, combined with a major shift towards accumulation of the late specific L1-IIIa mRNA (Fig. 7, lower panel, lanes 5 and 6). These effects were not observed with the Cα1K73M kinase inactive mutant (Fig. 7, lanes 7–8 and 11–12).

## Discussion

Here we show that the adenovirus L4-33K protein specifically associates with the nuclear DNA-PKcs in uninfected and adenovirus-infected cells. Further, we show that the L4-33K protein is highly phosphorylated by DNA-PK *in vitro* in a double stranded DNA-independent manner. Notably, DNA-PK appears to block the early to late switch in L1-52,55K/IIIa mRNA splicing suggesting that DNA-PK has an activity as a regulator of adenovirus MLTU alternative splicing. Further, we also show that L4-33K is phosphorylated by PKA and that PKA has an enhancing effect on L4-33K- activated L1-IIIa splicing. Taken together, our results show that both DNA-PK and PKA phosphorylate L4-33K but have distinct and opposite effects on the early to late shift in L1 alternative splicing.

Despite the fact that L4-33K contains multiple potential phosphorylation sites in its primary sequence, protein kinases that phosphorylate L4-33K have previously not been characterized [2], [40]. Identification of DNA-PK and PKA as L4-33K phosphorylating factors is our first successful attempts to characterize the interplay between L4-33K phosphorylation and gene expression regulation.

The purified L4-33K is an excellent substrate for DNA-PK in *in vitro* kinase assays. In contrast, L4-22K was phosphorylated to a much lower level suggesting that the primary DNA-PK phosphorylation sites resides in the unique C-terminal domain of L4-33K. This conclusion is supported by the observation that the L4-33Kds mutant protein was not efficiently phosphorylated by DNA-PK. Taken together, these data suggest that the ds domain contains the main residues phosphorylated by DNA-PK. Interestingly, both L4-33K and L4-22K are phosphorylated in the absence of dsDNA, which is the classical activator of DNA-PK (Fig. 4). Although unusual, other proteins are also phosphorylated by DNA-PK in the absence of dsDNA, like the forkhead transcription factor FoxA2 and the dysbindin-1 protein [41], [42]. In fact, the nuclear receptor co-activator thyroid hormone receptor-binding protein (TRBP) can itself activate DNA-PK in the absence of dsDNA [43]. In addition, an RNA-dependent phosphorylation mechanism has also been reported for the hnRNP C protein and the nuclear DNA helicase II protein (NDH II) [44]. These examples point to the variety of the mechanisms engaged in DNA-PK mediated protein phosphorylation.

L4-33K is also an excellent substrate for PKA phosphorylation in our *in vitro* phosphorylation assay. A bioinformatic search (NetPhosK server [45]) revealed that L4-33K has five putative PKA phosphorylation sites (data not shown), of which four are located within the unique C-terminal end. The fact that L4-33K and L4-22K only share the N-terminus supports our observation that L4-33K is a better PKA substrate compared to L4-22K. Out of the four predicted PKA phosphorylation sites in the C-terminal end, two are located within the functional ds domain. Identification of specific phosphorylated amino acids for both DNA-PK and PKA will need further investigation.

To this end, the specificity of L4-33K and DNA-PKcs interaction was verified in two different experimental systems (Figs. 2 and 3), supporting a functional association of these two proteins. Interestingly, our affinity purification experiments did not reveal the presence of the individual Ku proteins along with the DNA-PKcs. One possible explanation for this observation might be a suboptimal detection of the Ku proteins in our experimental system. However, DNA-PKcs can also phosphorylate substrate proteins in Ku-independent manner [13]. Therefore, it is also possible that the Ku proteins are not essential for stable L4-33K:DNA-PKcs complex formation. Two other adenoviral proteins, E4-ORF3 and E4-ORF6, also associate with DNA-PK. The linear adenovirus genome is recognized as a double-stranded break by the MRN DNA repair system, leading to viral DNA concatenation [46]. It has been shown that E4-ORF3 and E4-ORF6 binding to DNA-PK is important to block this genome concatenation [46], [47].

It is intriguing that adenovirus encodes for three proteins (E4-ORF3, E4-ORF6 and L4-33K), which all can target DNA-PK. From this perspective it is interesting to note that all three proteins appears to be regulators of adenovirus alternative splicing. Thus, here we show that DNA-PK blocks the early to late switch in adenovirus L1 alternative splicing (Fig. 5). We have previously shown that the E4-ORF3 and E4-ORF6 proteins function as alternative splicing factors *in vivo*; E4-ORF3 facilitating i-leader exon inclusion and E4-ORF6 favoring i-leader exon skipping [48]. Considering the known functions of these proteins it is possible that the individual proteins target DNA-PK in a temporal and spatial manner throughout the adenovirus life cycle.

Pre-mRNA splicing occurs in a co-transcriptional manner with splicing factors interacting with CTD in the RNAPII complex [49]. Interestingly, DNA-PK can phosphorylate the CTD at Ser2 and Ser7, pointing to a possible indirect role of DNA-PK in splicing [50]. Further, the regulatory Ku86 subunit of DNA-PK has been functionally associated with RNAP II elongation independently of DNA-PKcs [51]. In addition, Ku86 resembles the hnRNP family of splicing factors because of its high affinity to G-rich sequences [44]. These findings indicate a possible role for Ku86 also in pre-mRNA processing, maybe as an alternative splicing factor. Further experiments are needed to establish whether Ku86 plays a role in activation of the atypical L1-IIIa 3′ splice site late during infection.

We did not identify any PKA subunits in our GST-pull-down experiments. This could be due to the low abundance of PKA C in nuclear extracts compared to the whole cell [34]. If PKA C was pulled down, detection would be difficult in CBB stained gels due to several proteins of approximately the same size (40 kDa). We therefore conducted co-immunoprecipitations with HEK293T cells transfected with L4-33K-FLAG and PKA Cα1, but we were not able to detect any interaction (data not shown). That PKA does not stably interact with L4-33K was not entirely unexpected, since a direct interaction between PKA and its substrate proteins has been described for only a few of the over hundred characterized PKA substrates [52].

Two of the four predicted PKA phosphorylation sites in the C-terminal end of L4-33K are located within the functional important ds region of L4-33K, suggesting a possible connection between the massive effect of PKA on L4-33K activated L1-IIIa mRNA splicing (Fig. 7), and thereby further activation of late gene expression. This correlates well with previous reports showing that Ad5 replication is sensitive to PKA activity, as the adenylyl cyclase activator forskolin increase and the PKA inhibitor H-89 decrease replication [53]. Interestingly, the same report shows that the Adeno-associated virus (AAV2), which is dependent upon a helper virus for a productive infection, actually inhibit adenovirus replication by expression of the two viral proteins Rep 78 and Rep 52 that interact with PKA C via a domain similar to that of its endogenous nuclear inhibitor, PKI.

PKA also had a marked effect on total L1 mRNA accumulation, suggesting a possible function of the protein kinase on MLP transcription. Since we analyzed total RNA in this experiment (Fig. 7) this effect most likely reflects a direct effect of PKA on transcription, although we cannot exclude that PKA affects RNA stability. This effect on transcription was not expected since the MLP driving expression from the reporter plasmid does not contain identified CRE binding sites [54]. It is known that PKA C directly activates other transcription factors, for instance p65 Rel of the NFκB-complex [55] and Heat shock transcription factor 1 (HSF1) [25]. Whether any of these factors regulate the MLP remains to be investigated. Collectively, our results suggest a novel regulatory role of PKA in L1 alternative splicing. We have previously shown that L4-33K activates splicing via weak 3′splice sites, whether the stimulatory effect seen also is dependent on these sequence elements or others needs to be investigated. These data together suggests two roles of PKA during MLTU gene expression; a transcriptional activation of MLP and a major stimulatory effect on L4-33K activated L1-IIIa pre-mRNA splicing.

Other roles for PKA during adenoviral gene expression have been reported earlier. Already during the initial phase of the infection PKA is involved in microtubule-mediated nuclear targeting of the virus [56]. Furthermore, it has been demonstrated that the E1A_12S_ protein in fact functions as a viral AKAP that redistributes the RIIα subunit to the nucleus where it is involved in activation of the E2 promoter [57].

Interestingly, a link between DNA-PK and PKA has been reported for the Epstein Barr virus (EBV) protein EBNA-LP, which is a co-activator of EBNA-2-mediated transcription. EBNA-LP binds to several cellular proteins, including DNA-PKcs and homologous to AKAP95 (HA95) [58]. It was later shown that the PKA C subunit, but not the R subunit, is associated with HA95 and EBNA-LP [24]. It is therefore suggested that that in EBV infected cells, HA95 may function as a scaffold for EBNA-LP, DNA-PKcs, PKA C and other proteins involved in virus-mediated transcriptional activation. Whether something similar also is the case during adenovirus infection remains to be elucidated. Of relevance for our study, it is interesting to note that we have previously shown that HA95 also is involved in regulation of adenovirus E1A pre-mRNA splicing [34].

In light of this and previous reports on viral interaction partners of PKA and DNA-PKcs at different stages of infection, it is tempting to speculate that various signaling complexes consisting of both cellular and viral proteins are important for regulation of the adenoviral life cycle. Such complexes containing counteracting proteins, such as DNA-PKcs and PKA Cα1, may contribute to the diversity of the highly coordinated spatial and temporal regulation of adenoviral gene expression.

In conclusion, this is the first report where the phosphoprotein L4-33K is connected to any kinase and here we present two cellular protein kinases with opposite effects on L4-33K function; DNA-PK as an inhibitor and PKA as an activator of L1-IIIa mRNA splicing. To the best of our knowledge this is the first demonstration that DNA-PK has a function as a regulator of RNA splicing. Collectively, our data suggests a novel interplay between the DNA-PK/PKA and L4-33K in regulation of adenovirus late alternative splicing.

## Materials and Methods

### Plasmids

For bacterial expression, cDNAs of L4-33K, L4-33Kds and L4-22K were cloned into pET24a (Novagen) expression vector producing L4-33K and L4-22K proteins containing carboxyl-terminal His-tags [2], [3]. pGST-L4-33K plasmid was generated by recloning L4-33K cDNA from pET24a-L4-33K [2] into pGEX-3× plasmid (Pharmacia). Plasmid pGST-p53 was used for expression of GST-p53 protein and has been described elsewhere [59]. Plasmids encoding full-length PKA Cα1 and the catalytically inactive mutant Cα1K73M in the mammalian expression vector pEF-DEST 51 have been described previously [34], [60]. Plasmid encoding L4-33K in the mammalian expression vector pcDNA3 (Invitrogen), and the reporter plasmid, pTripL1,L2 have previously been described [2], [3].

### Protein purification

pGST-L4-33K and pGST-p53 were expressed in Escherichia coli BL21 (DE3), and DH5, respectively. The expression of the GST-L4-33K and GST proteins were induced with 0.1mM IPTG for 3 hours at 37°C. The cells were lysed in lysis buffer (20 mM Hepes-KOH pH 7.9, 300 mM KCl, 0.05% Triton X-100) supplemented with protease inhibitor (Complete Mini EDTA-free, Roche Applied sciences) and sonicated with high output 4×30 s on a Bioruptor® sonicator (Diagenode). Soluble lysates were incubated with Glutathione Sepharose™ 4B (GE Healthcare) and GST-tagged proteins were recovered by end-over-end rotation for 2 h at 4°C. Beads were sedimented and washed 5 times 1 ml in lysis buffer. Bound proteins were eluted with Buffer D (20 mM Hepes-KOH pH 7.9, 20% glycerol, 0.1 M KCl, 0.2 mM EDTA pH 8, 0.5 mM dithiotreitol (DTT)) supplemented with 5 mM of reduced glutathione (Sigma). Eluates were dialyzed against Buffer D and stored at −80°C. Protein concentration was determined by using bovine serum albumin (BSA) as the standard on a Coomassie Brilliant Blue (CBB) stained SDS-PAGE gel. Recombinant his-tagged L4-33K proteins used in this study were purified by Nickel column chromatography as previously described [2].

### GST pull-down assay

One mg of HeLa cell nuclear extract (HeLa-NE) or adenovirus late infected nuclear extract (Ad-NE) [61] was mixed with 5 µg of GST or GST-L4-33K proteins in a total volume of 500 µL of 60% Buffer D. All protein samples were cleared from precipitated proteins by centrifugation (16060× g, 3 min at 4°C). Following addition of 10 µL of packed Glutathione Sepharose™ 4B samples were incubated for 2 h with end-over-end rotation at 4°C. The beads were washed five times with 60% Buffer D before resuspended in Laemmli buffer [62]. The samples were heated at 95°C for 5 min. and separated on SDS-PAGE gel (any kD™, Biorad). The gels were stained with colloidal CBB. The visible protein bands were cut out and analyzed by mass spectrometry.

### Construction of a recombinant adenovirus expressing a FLAG-tagged L4-33K protein

Transfer plasmid pShuttleTetLac-Ad5-L4-33K was constructed by isolating the L4-33K encoding region from pcDNA3-L4-33K plasmid [3] by digestion with Nco1, ligation of Bcl-8 linker (CTGATCAG) and recleavage with Bcl1. The Bcl1 fragment was cloned into the BamH1 site in the pShuttleTetLac-Bam vector [63]. pShuttleTetLac-Ad5-L4-33K was reconstructed into a modified recombinant AdEasy virus encoding for the Tet-ON transcriptional activator protein inserted into the E3 region [63].

### Cells, infections and transfections

HEK293 cells (ATCC) were grown on 60 mm plates in Dulbecco's modified eagle medium (DMEM, Invitrogen) supplemented with 10% newborn calf serum (NCS) and 1% penicillin-streptomycin (PEST, Invitrogen), infected at 10 fluorescence forming units (FFU) per cell of recombinant AdEasy-L4-33K or AdEasy-GFP viruses. MO59K (DNA-PK positive) and MO59J (DNA-PK negative) cells [64]–[66] were grown in DMEM/F12 media (Invitrogen) supplemented with 10% foetal calf serum and 1% PEST. The MO59K and MO59J cells were infected at 10 FFU/cell of wild type Ad5 virus. HEK293T cells (ATCC) were maintained at 37°C in humidified air with 5% CO_2_ in RPMI 1640 (Sigma-Aldrich) supplemented with 10% foetal bovine serum (Sigma-Aldrich), 1% non-essential amino acids (GibcoBRL), 1% L-glutamine (Sigma-Aldrich), 1% sodium pyruvat (GibcoBRL) and 1× PEST (Sigma-Aldrich). The cells were transfected with Fugene HD (Roche) according to the manufacturer's protocol. Briefly, 6 µL Fugene HD (Roche) and a total of 2 µg DNA were mixed in OPTIMEM, added to each well and incubated for 24 hours. For PKA/L4-33K S1 analysis, cells were co-transfected with the pTripL1,L2 reporter plasmid (0.8 µg) and pcDNA3-L4-33K (0.1–0.6 µg) and/or pEF-DEST 51 Cα1 (0.1–0.6 µg)/pEF-DEST 51 Cα1K73M (0.1–0.6 µg)/pEF-DEST 51 empty vector. For the L4-33K dephosphorylation assay, the cells were transfected with 2 µg pcDNA3-L4-33K. For immunoprecipitation and 2D-gel analysis subconfluent HEK293 cells (ATCC) were transfected using the calcium phosphate co-precipitation method [67]. For 150-mm plates a total of 25 µg of DNA was used.

### Small-scale nuclear extract preparation

HEK293 cells (ATCC) were grown on four 150 mm plates in DMEM (Invitrogen) supplemented with 10% NCS and 1% PEST, infected at 10 FFU/cell of recombinant AdEasy-L4-33K or AdEasy-GFP virus. Nuclear extract was prepared 20 hpi by, resuspending cell pellets in 3× packed cell volume Buffer A (10 mM Hepes-KOH pH 7.9, 1.5mM MgCl_2_, 10 mM KCl, 0.5 mM DTT). After 10 minutes of cell swelling, the lysates were homogenized using a 7 mL dounce homogenizer with a tight pestle until approximately 90% of the cells were lysed. Nuclei were separated by centrifugation, resuspended in 1× packed nuclei volume Buffer C (20 mM Hepes-KOH pH 7.9, 25% (v/v) glycerol, 600 mM KCl, 0.2 mM EDTA pH 8, 0.5 mM DTT) and further homogenized with 10 strokes through a 23G needle. Finally, the nuclear lysates were incubated for 30 minutes with gentle mixing every 5 min. Nuclear extract was cleared by centrifugation and dialysed against Buffer D using 7 kDa cut off cups.

### Immunoprecipitation

NE was diluted to a final concentration of 60% Buffer D in a total volume of 1 mL and pre-cleared by incubation with Sepharose® CL-4B (GE Healthcare) and end-over-end rotation for 1 h at 4°C. Pre-cleared extracts were incubated at 30°C for 5 min before 20 µL of packed anti-FLAG® M2 affinity gel (Sigma) was added and incubation continued for 2 h by end-over-end rotation at 4°C. Beads were sedimented on ice for 25 min and washed four times with 60% Buffer D. Bound proteins were eluted with 150 ng/µL 3×FLAG peptide (Sigma) in TBS (50 mM Tris-Cl, 150 mM NaCl, pH 7.5) by gently shaking the tubes for 1 h at 4°C. The eluates were mixed with Laemmli buffer, incubated 96°C for 5 min and analyzed on 12% SDS-PAGE. Gels were stained with colloidal CBB and bands were cut out and analyzed by mass spectrometry.

### Immunoprecipitation and 2D-gel analysis

Forty hours post transfection, cells were washed in PBS and lysed with IsoB-NP40 (10 mM Tris-HCl [pH 7.9], 0.15 M NaCl, 1.5 mM MgCl_2_, 0.5% Nonidet P-40) supplemented with protease inhibitor complete mini EDTA-free [Roche]. The lysates were cleared by centrifugation, adjusted to equal protein concentration, and incubated with anti-FLAG® M2 affinity gel (Sigma) for 2 h at 4°C. Beads were collected by sedimentation for 20 min on ice and washed 3 times with IsoB-NP40. Bound proteins were eluted with 150 ng/µL 3×FLAG peptide (Sigma) in TBS by gently shaking the tubes for 4 h at 4°C. Eluted proteins were precipitated by addition of 8 volumes of acetone. Proteins were collected by centrifugation and mixed with Laemmli buffer, incubated 96°C for 5 min and analyzed by 2D-gel electrophoresis by first dimension separation between pH 3–11, and second dimension separation on 12.5% SDS-PAGE gel. Gels were stained with colloidal CBB and bands were cut out and analyzed by mass spectrometry.

### S1 protection assay

Cytoplasmic or total RNA (Rneasy kit, Qiagen) was harvested 24 h post infection or transfection as previously described [2] or according to the manufacturers instructions. Five µg of RNA was used for the S1 analysis with an L1 probe. Conditions and preparation of the DNA probe was as previously described [3]. The statistical analysis was performed with paired student's t-test in Prism GraphPad.

### *In vitro* phosphorylation

The DNA-PK kinase assay was performed according to the manufacturers instructions (Promega) but omitting EGTA in the reaction buffer. Briefly, 0.3–0.5 µg of substrate proteins were mixed with reaction buffer resulting in the final concentration of 50 mM Hepes-KOH pH 7.9, 0.1 M KCl, 10 mM MgCl_2_, 1 mM DTT, 0.1 mM EDTA pH 8, 0.2 mM ATP, 80 µg/mL BSA, 2 µCi of γ^32^P-ATP and when noted 10 µg/mL linear double-stranded DNA (dsDNA; HpaII cleaved and phenol-chloroform purified pUC19 (Fermentas) plasmid DNA). All components, except DNA-PK, were mixed and incubated 3 min at 30°C before 10 U of DNA-PK was added and the reaction further incubated for 10 min at 30°C. Reactions were stopped by addition of Laemmli buffer, incubated at 95°C for 5 min before proteins were separated on 12% SDS-PAGE gel, followed by CBB staining. The incorporation of γ^32^P-ATP was analyzed by PhosphorImager (BioRad) scanning. PKA *in vitro* phosphorylation assay was performed as previously described [34]. Briefly, 2.5 ng active or heat-inactivated (10 minutes at 70°C) PKA Cα1 (Invitrogen) were mixed with *in vitro* phosphorylation buffer (10 mM potassium phosphate pH 7.4, 1 mM EDTA, 10 mM magnesium acetate and 1 µCi γ^32^P-ATP). 0.25 µg purified L4-33K or L4-22K was added to a total volume of 20 µl. The reactions were incubated at 30°C for 30 minutes on slushy ice for one hour and stopped by adding SDS loading dye and boiled 5 minutes. The proteins were resolved by SDS-PAGE and analyzed by CBB staining and autoradiography.

### Dephosphorylation assay

Twenty-four hours after transfection, cells were washed in PBS and lysed by sonication in IP buffer (150 mM NaCl, 50 mM Tris pH 7.5, 0.5% Triton X-100, 1× protease inhibitor cocktail (Sigma), 1 mM PMSF and 1 mM Na_3_VO_4_). The lysates were cleared by centrifugation and adjusted to equal protein concentration (2 µg protein/µl) by the Bradford method (BioRad). The lysates were precleared with Dynabeads Protein G (Invitrogen, 1∶10) and incubated over night at 4°C with 2.5 µg rabbit anti-FLAG antibody (Sigma). Dynabeads Protein G (1∶10) were added and incubated for 1 hour at 4°C. The samples were washed three times with IP buffer. The dephosphorylation method was done as previously described [68]. Briefly, the magnetic Dynabeads were added to 100 units of alkaline phosphatase (Sigma) in 5 mM Tris pH 7.9, 10 mM NaCl, 1 mM MgCl_2_ and 0.1 mM DTT and treated for 30 minutes at 30°C or left untreated on ice. Reaction was stopped by addition of SDS loading dye and boiled for 5 minutes before proteins were separated by SDS-PAGE (15%) and transferred to PVDF membranes. Immunoreactive proteins were detected by anti-FLAG M2 (Sigma, 1∶1000).
